# Optimal D-Shaped Toolpath Design for Minimizing X-Axis Servo Following Error in Turning the Off-Axis Optical Surfaces

**DOI:** 10.3390/ma18184343

**Published:** 2025-09-17

**Authors:** Baohua Chen, Quanying Wu, Yunhai Tang, Fei Wang, Junliu Fan, Xiaoyi Chen, Haomo Yu, Yi Sun

**Affiliations:** 1 Key Laboratory of Efficient Low-Carbon Energy Conversion and Utilization of Jiangsu Provincial Higher Education Institutions, School of Physical Science and Technology, Suzhou University of Science and Technology, Suzhou 215009, China; chenbaohua@usts.edu.cn (B.C.);; 2 Computing Science and Artificial Intelligence College, Suzhou City University, Suzhou 215104, China; 3 Suzhou Key Laboratory of Multi-Modal Data Fusion and Intelligent Healthcare, Suzhou City University, Suzhou 215104, China; 4 Suzhou Mason Optical Co., Ltd., Suzhou 215127, China; 5 Graduate Practice Station in Soochow Mason Optics Co., Ltd., Suzhou 215127, China

**Keywords:** slow tool servo, D-shaped toolpath, high-precision turning, servo following error

## Abstract

In the slow tool servo (STS) turning technology for optical lenses, the D-shaped toolpath can improve the quality of the optical surfaces of off-axis aspheric and cylindrical microlens arrays. However, the traditional D-shaped toolpath has the problem of excessive servo following error in the X-axis. To address this issue, the projection of the D-shaped toolpath in the XZ plane is divided into a cutting zone and a transition zone. In the transition zone, an equation system based on continuity constraints (surface height, feed-rate, acceleration) is established. By solving this system of equations, a toolpath can be obtained along which the feed-rate of the X-axis varies smoothly. An example shows that the acceleration of the X-axis of the lathe is reduced by 84% compared to the traditional D-shaped toolpath. In the XZC interpolation mode, the spindle velocity of the C-axis changes smoothly. An off-axis spherical surface and an integral mirror have been machined using the optimized D-shaped toolpath. The X-axis servo following error of the lathe during processing is within 7 nm, and the surface shape accuracy reaches 0.361λ at 632.8 nm. This method enables high-precision processing of off-axis curved surfaces and cylindrical arrays.

## 1. Introduction

Non-rotationally symmetric optical components, such as gratings [1,2,3], off-axis lenses [4,5,6,7,8] and microlens arrays [9,10,11,12], play a significant role in spectral imaging, optical three-dimensional display and beam shaping. The processing of complex freeform surface lenses mainly relies on precision turning technology. The material properties, feed-rate, and turning methods all influence the quality of the lens surface. The precision manufacturing of non-rotationally symmetric optical lenses is usually accomplished by techniques such as Fast Tool Servo (FTS), micro-milling, fly-cutting, precise grinding, and Slow Tool Servo (STS). In FTS technology, through innovative hinge structure [13], dual correction of toolpath [14], dynamic path optimization [15], adaptive sampling [16] and other strategies, the machining accuracy of optical surfaces has been significantly improved. However, FTS technology still faces common challenges that need to be addressed, such as the efficiency of control point management and the occurrence of tool interference. In terms of micro-milling technology, the Wavefront Error Correction strategy is optimal in optical parameters [17], the tilt strategy stands out in surface morphology robustness [18], and the trajectory model leads in model prediction and multi-functionality [19]. However, the equipment cost of microstructure milling technology is high and its efficiency needs to be improved. The fly-cutting technology can be directly applied to process nickel-based micro-optical waveguide molds [20], and also achieve deterministic nanostructure processing on complex curved surfaces [21]. However, the complexity of system debugging and the dependence on tools remain challenges for engineering applications. In the ultra-precision grating turning process, the pulsed spindle vibration has a dominant influence on the surface topography [22]. In the high-precision grinding of freeform surface optical components, the toolpath for the subsequent grinding step is corrected by comparing the measured surface topography with the nominal model, thereby enhancing the overall processing accuracy [23]. Nevertheless, there remains significant potential for further improvements in both the accuracy of correction methods and their adaptability. STS is suitable for turning complex curved surfaces. The spatial helical normal projection technique is used to process large slope curved surfaces [24]. Adaptive sampling and smooth feed technology can efficiently process large-aperture optical surfaces [25]. A toolpath planning method based on NURBS curvature analysis, through surface reconstruction and curvature classification, resolves the path distortion problem of the traditional Archimedean spiral method in free-form surface machining [26]. The advantage of the method for directly generating toolpaths based on point clouds lies in avoiding fitting errors and enhancing the stability of machine tools [27]. The Z-direction tool compensation method avoids the oscillation of the lathe’s X-axis during the machining process [28,29,30].

New optical surfaces with linear arrangement structure, such as the integral mirrors composed of a cylindrical array and the sinusoidal surface or the linear grating reflective surface, have abrupt changes on their surfaces. If the single-point diamond turning machine adopts the traditional helical toolpath, when the height undergoes a sudden change, the velocity of the Z-axis movement will change dramatically [25], which will lead to an inability to respond promptly and thus affect the processing accuracy. Some commercial software (such as DIFFSYS 5.1, etc.) can generate a D-shaped toolpath composed of arcs and straight lines. The direction of the toolpath is parallel to the boundary where the surface height undergoes a sudden change, thereby avoiding sudden changes in the Z-axis velocity. The D-shaped toolpath can be used to process both convex surfaces and concave lenses. It increases the spindle velocity of the C-axis and reduces the servo following error within a certain range. However, at the junction between the arc and the straight line, the feed-rate of the X-axis undergoes a sudden change. In the XZC interpolation mode, the spindle velocity of the C-axis will experience sudden changes, which will lead to significant servo following errors and thus affect the machining accuracy.

To address the issue of large servo following errors in the STS processing, an optimized design method is proposed for the D-shaped toolpath. The proposed method reduces the response frequency of the Z-axis while controlling the feed-rate and acceleration of the X-axis, effectively reducing the servo following errors. Additionally, this method improves processing efficiency by piecing multiple cutting zones within a circular toolpath.

## 2. Methods

**Figure 1:**
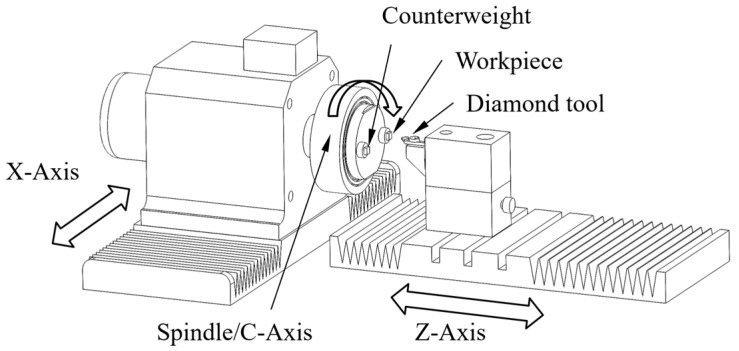
Schematic diagram of single-point diamond turning for off-axis surfaces.

Figure 1 shows a schematic diagram of an ultra-precision lathe with STS. In STS turning, off-axis surfaces are machined by synchronizing the movements of the X and Z axes with the rotation of the C axis. In order to counteract the unbalanced torque caused by the rotation of the workpiece and reduce the vibration of the spindle, counterweight is installed.

**Figure 2:**
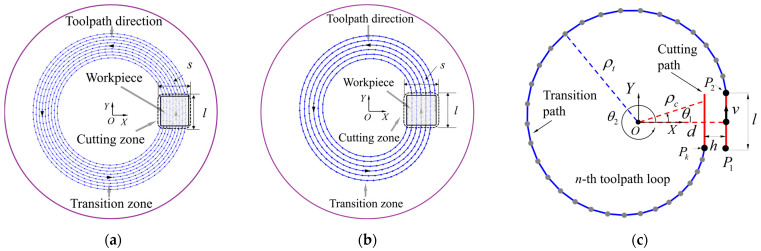
Projection of D-shaped toolpath to the XY plane: (**a**) schematic diagram of the traditional D-shaped toolpath; (**b**) schematic diagram of the optimized D-shaped toolpath; (**c**) the structure of the optimized D-shaped toolpath.

In the process of single-point diamond turning for optical surfaces, the toolpath has a significant impact on the machining result. The three-axis STS toolpath projected onto the XY plane (also known as the *ρ*-*θ* plane) is called the projected toolpath. The entire projected toolpath is divided into a cutting zone and a transition zone within the range of polar angle 0~2*π*. Figure 2a shows the schematic diagram of the traditional D-shaped toolpath. The cutting zone is a rectangle with a length of *l* and a width of *s*. The workpiece is contained within the cutting zone. The toolpath in the cutting zone and the toolpath in the transition zone of the traditional D-shaped toolpath exhibit first-order continuity. Figure 2b shows the optimized D-shaped toolpath. In the transition zone, the tool smoothly moves from the end of the previous cutting toolpath to the starting point of the next cutting toolpath. Figure 2c is a schematic diagram of a single optimized D-shaped toolpath. The red lines are the toolpaths within the cutting zone, and the blue curves are the toolpaths within the transition zone. According to the right triangle geometric relationship, there are

(1) $$\theta_{1}=\arctan\frac{l}{2d},$$

(2) $$\theta_{2}=2\pi -\arctan\frac{l}{2(d-h)},$$

where *l* is the length of the cutting toolpath, *d* = *d_e_−nh* represents the distance from the straight toolpath to the rotation center of the C-axis, *h* is the toolpath spacing, *d_e_* is the distance from the outermost edge of the cutting zone to the C-axis rotation center. *θ*_2_−2π and *θ*_1_ are the starting and ending angles of the cutting toolpath, respectively.

As can be seen from Figure 2, *O* is the center of C-axis rotation, and the cutting toolpath is a series of mutually parallel line segments. In the *n*th cycle, the polar angle *θ* increases from 2(*n*−1)*π* to 2*nπ*. As shown in Figure 2c, the polar radius of the cutting zone’s toolpath is

(3) $$\rho_{c}(n,\theta )=d\sec\theta \;\;\;(\theta_{2}-2\pi \leq \theta \leq \theta_{1}),$$

Let the polar radius of the transition toolpath be *ρ_t_*(*n*, *θ*), then

(4) $$\rho_{t}(n,\theta )=\sum_{m=0}^{5}c_{m}\theta^{m}\;\;\;(\theta_{1}\leq \theta \leq \theta_{2}),$$

where *c_m_* is a polynomial coefficient. Analyzing Equation (4), *ρ_t_* is characterized by a fifth-degree polynomial about *θ*.

Assuming that the spindle velocity ω of the C-axis is a constant value, the feed-rate *v_ρ_* and acceleration *a_ρ_* of the X-axis can be expressed as follows:

(5) $$v_{\rho }=\frac{\partial \rho }{\partial \theta }\cdot \omega ,$$

(6) $$a_{\rho }=\frac{\partial^{2}\rho }{\partial \theta^{2}}\cdot \omega^{2},$$

where *ρ* is the polar radius of the projected toolpath to the XY plane.

In order to ensure the continuity of the polar radius at the junction of the cutting zone and the transition zone, and to ensure that the rate of change in the polar radius with respect to the polar angle is smooth, the polar radius is subject to the following constraints:

(7) $$\rho_{t}(n,\theta_{1})=\rho_{c}(n,\theta_{1}),$$

(8) $$\rho_{t}(n+1,\theta_{2})=\rho_{c}(n,\theta_{2}),$$

(9) $$\frac{\partial \rho_{t}(n,\theta )}{\partial \theta }{|}_{\theta =\theta_{1}}=\frac{\partial \rho_{c}(n,\theta )}{\partial \theta }{|}_{\theta =\theta_{1}},$$

(10) $$\frac{\partial \rho_{t}(n+1,\theta )}{\partial \theta }{|}_{\theta =\theta_{2}}=\frac{\partial \rho_{c}(n,\theta )}{\partial \theta }{|}_{\theta =\theta_{2}},$$

(11) $$\frac{\partial^{2}\rho_{t}(n,\theta )}{\partial \theta^{2}}{|}_{\theta =\theta_{1}}=\frac{\partial^{2}\rho_{c}(n,\theta )}{\partial \theta^{2}}{|}_{\theta =\theta_{1}}=0,$$

(12) $$\frac{\partial^{2}\rho_{t}(n+1,\theta )}{\partial \theta^{2}}{|}_{\theta =\theta_{2}}=\frac{\partial^{2}\rho_{c}(n,\theta )}{\partial \theta^{2}}{|}_{\theta =\theta_{2}}=0,$$

Equations (7)–(12) constitute a linear system of equations for the polynomial coefficients *c_m_*, and the system has been arranged in the following matrix form:

(13) $$\left(\begin{array}{llllll} \theta_{1}^{5} & \theta_{1}^{4} & \theta_{1}^{3} & \theta_{1}^{2} & \theta_{1} & 1\\ \theta_{2}^{5} & \theta_{2}^{4} & \theta_{2}^{3} & \theta_{2}^{2} & \theta_{2} & 1\\ 5\theta_{1}^{4} & 4\theta_{1}^{3} & 3\theta_{1}^{2} & 2\theta_{1} & 1 & 0\\ 5\theta_{2}^{4} & 4\theta_{2}^{3} & 3\theta_{2}^{2} & 2\theta_{2} & 1 & 0\\ 20\theta_{1}^{3} & 12\theta_{1}^{2} & 6\theta_{1} & 2 & 0 & 0\\ 20\theta_{2}^{3} & 12\theta_{2}^{2} & 6\theta_{2} & 2 & 0 & 0 \end{array}\right)\left(\begin{array}{c} c_{5}\\ c_{4}\\ c_{3}\\ c_{2}\\ c_{1}\\ c_{0} \end{array}\right)=\left(\begin{array}{l} \rho_{c}(n,\theta_{1})\\ \rho_{c}(n,\theta_{2})\\ \frac{\partial \rho_{c}(n,\theta )}{\partial \theta }{|}_{\theta =\theta_{1}}\\ \frac{\partial \rho_{c}(n+1,\theta )}{\partial \theta }{|}_{\theta =\theta_{2}}\\ 0\\ 0 \end{array}\right),$$

Since the above system of equations consists of six equations, it is optimal to choose a fifth-degree polynomial as the function to describe the transition zone of the trajectory. Solving the system of Equation (13) yields the polynomial coefficients *c_m_*, thus obtaining the transition toolpath.

**Figure 3:**
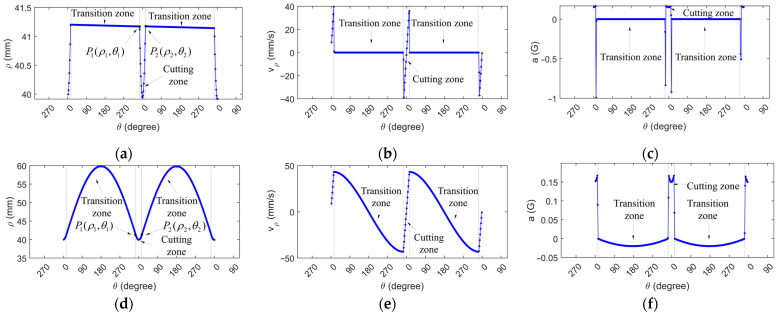
The distribution of the polar radius of the D-shaped tool path, as well as the velocity and acceleration of its variation: (**a**) polar radius variation in the traditional D-shaped toolpath; (**b**) rate of polar radius variation in the traditional D-shaped toolpath; (**c**) acceleration of polar radius variation in the traditional D-shaped toolpath; (**d**) polar radius variation in the optimized D-shaped toolpath; (**e**) rate of polar radius variation in the optimized D-shaped toolpath; (**f**) acceleration of polar radius variation in the optimized D-shaped toolpath.

In the following section, the characteristics of the optimized toolpath are illustrated through an example. Assume that the length *l* and width *s* of the cutting zone are both equal to 20 mm. The distance from the center of the cutting zone to the center of the C-axis will affect the velocity and acceleration of the X-axis. In this instance, its value is 30 mm. Figure 3a shows the variation in the polar radius of the traditional toolpath in two cycles. In the transition zone, the polar radius remains nearly constant, whereas in the cutting zone, it first decreases rapidly, then increases, and ultimately ceases to grow abruptly. As shown in Figure 3d, the polar radius in the optimized toolpath smoothly transitions from the cutting zone to the transition zone.

To facilitate a quantitative comparison of the X-axis feed-rate and acceleration, the spindle velocity of the C-axis is set as a constant value of 40 rpm. The rate of variation in the polar radius is calculated according to Equation (5). It reflects the feed-rate of the X-axis of the lathe. It can be seen from Figure 3b that the feed-rate of the traditional D-shaped toolpath changes abruptly, while in Figure 3e, the feed-rate in the optimized toolpath changes continuously.

The acceleration variation in the polar radius with respect to the polar angle is calculated according to Equation (6). The x-axis acceleration of the traditional D-shaped toolpath is shown in Figure 3c. It suddenly increases to approximately one times the gravitational acceleration at the boundary of the cutting zone. In contrast, the X-axis acceleration of the optimized D-shaped toolpath is shown in Figure 3f. The maximum acceleration of the X-axis is controlled within the cutting zone. Under the current settings, the acceleration of the X-axis of the lathe along the optimized D-shaped toolpath is 16% of that along the traditional D-shaped toolpath. This means that the acceleration of X-axis along the optimized D-shaped toolpath has been reduced by approximately 84%.

In the XZC interpolation mode, the spindle velocity of the C-axis changes along with the movement of the X-axis. The following situation can be easily deduced in reverse. In traditional tool path planning, when the feed-rate of the X-axis is maintained constant, the spindle velocity of the C-axis exhibits significant fluctuation. However, in the optimized tool path planning, the spindle velocity of the C-axis is more stable.

**Figure 4:**
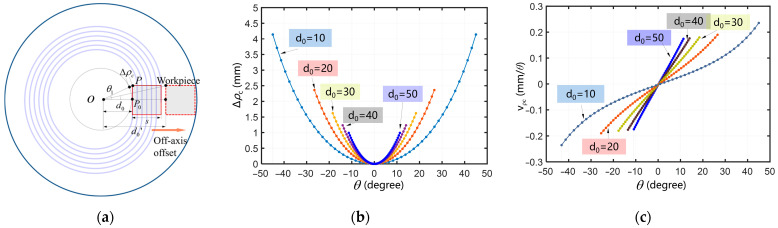
Diagram of workpiece offset distance, change in polar radius Δ*ρ_c_* in the cutting zone and feed-rate: (**a**) schematic diagram of workpiece offset distance; (**b**) variation curve of polar radius difference Δ*ρ_c_*; (**c**) variation curve of feed-rate v*_ρc_*.

In Figure 4a, *P*_0_ is the center of the left edge of the cutting zone, *P* is the point on the upper left edge of the cutting zone. Here, the distance from *P*_0_ to the center of the C-axis is referred to as the off-axis distance *d*_0_. The maximum change in the polar radius in the cutting zone is Δ*ρ_c_* = *ρ_op_*−*d*_0_. Here, *ρ_op_* refers to the polar radius at point P. Figure 4b shows the change in polar radius Δ*ρ_c_* in the cutting zone as a function of the change in off-axis distance *d*_0_. When the workpiece moves from the square formed by solid lines to the one formed by dashed lines, both Δ*ρ_c_* and v*_ρc_* will change. The greater the off-axis distance *d*_0_, the smaller the Δ*ρ_c_*. Figure 4c shows the feed-rate v*_ρc_* of the polar radius in the cutting zone under different off-axis distances *d*_0_. Clearly, as the off-axis distance *d*_0_ increases, the variation curve of v*_ρc_* becomes increasingly linear. If the curve of feed-rate v*_ρc_* is a straight line, it indicates that the acceleration of the X-axis of the lathe is constant. The more stable the acceleration value of the X-axis of the lathe is, the smaller the error of the lathe operation will be.

In conclusion, by adopting the proposed method, the maximum acceleration of the X-axis of the lathe is within the cutting zone. When the maximum angle in the cutting zone is less than 20 degrees, v*_ρc_* basically shows a linear change, and then the X-axis of the lathe can achieve stable feed-rate. The maximum travel of the X-axis of the lathe is D. In actual processing, if the following conditions are met, the servo following error of the lathe can be controlled within a smaller range.

(14) $$\left\{\begin{array}{l} \arctan(\frac{l}{2d_{0}})\leq 20^{\circ }\\ d_{0}+s\leq \frac{D}{2} \end{array}\right.,$$

**Figure 5:**
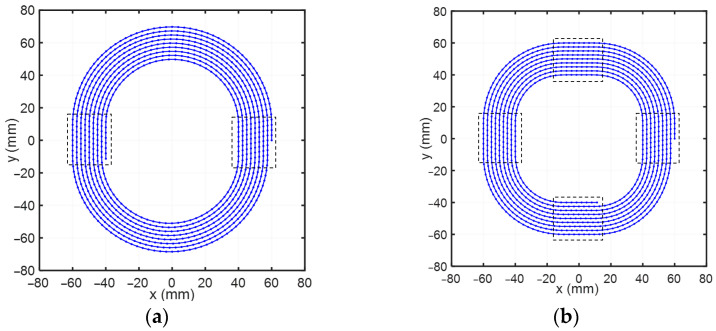
Multiple cutting tasks: (**a**) two cutting zones; (**b**) four cutting zones.

As shown in Figure 5a,b, within one cycle, two or four cutting zones can be set. This enables multiple cutting tasks to be accomplished simultaneously, significantly enhancing the cutting efficiency. The transition toolpath between adjacent two cutting zones is calculated based on the aforementioned equations.

## 3. Experiment and Discussion

**Figure 6:**
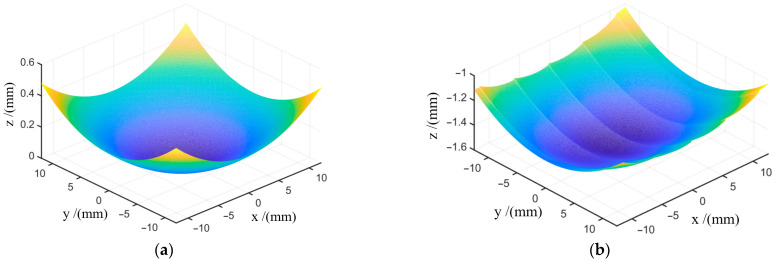
Spherical surface and integral mirror: (**a**) spherical surface; (**b**) integral mirror.

To test the effectiveness of the optimized D-shaped toolpath, a spherical surface and an integral mirror were processed. The spherical surface is a concave surface shown in Figure 6a with a curvature radius of 300 mm. The spherical surface can be easily tested using an interferometer. The integral mirror is formed by combining several curved cylindrical surfaces, as shown in Figure 6b. It has the function of splitting and converging light beams, so it can shape a Gaussian beam into a linear light spot. The apertures of both the spherical surface and the integral mirror are square shapes with a side length of 20 mm.

**Figure 7:**
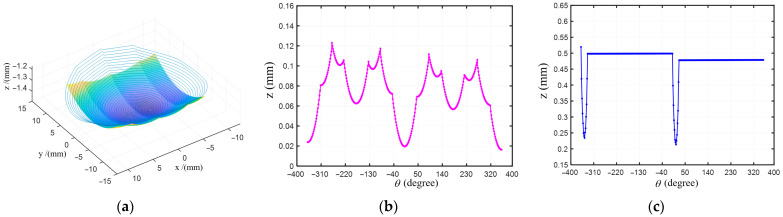
Comparison of spiral toolpath and D-shaped toolpath: (**a**) the surface of the integral mirror and spiral toolpath; (**b**) the z value on the spiral toolpath; (**c**) the z value on the D-shaped toolpath.

If the integral mirror is processed with the spiral toolpath as shown in Figure 7a, the Z-axis travel of the lathe within one revolution period is as shown in Figure 7b. Sharp peaks appear in the Z-axis travel at the junction of two adjacent cylindrical surfaces. This leads to extremely discontinuous movement of the Z-axis. Even if the Z-axis of the lathe has a relatively high response frequency, it is still difficult for the three axes of the lathe to remain synchronized. In the XZC interpolation mode, this will cause the C-axis to experience jolts during the processing of the integral mirror.

The D-shaped toolpath can significantly reduce the amplitude of jumps of the Z-axis of the lathe. The z value in the transition zone is obtained through linear interpolation based on the difference between the end and start points of adjacent cutting toolpaths within the cutting area (for example, points *P_k_* and *P*_2_ in Figure 2c). The calculation formula is as follows:

(15) $$z_{t}(n,\theta )=z_{p}(n,\theta_{1})+\frac{\theta -\theta_{1}}{\theta_{2}-\theta_{1}}\left[z_{p}(n+1,\theta_{2})-z_{p}(n,\theta_{1})\right],$$

Here, *n* represents the serial number of the current toolpath cycle.

As shown in Figure 7c, the value of the Z-axis of the lathe has a relatively small fluctuation. The tool position point is obtained by performing the tool nose radius compensation in the normal direction of the XZ plane. At this time, due to the non-rotational symmetry of the surface, the projected polar radius of the compensated tool position point will experience nonlinear fluctuations [28]. The expected polar radius of this tool position point is corrected to be linear by using the stable X-axis difference iterative method [30].

In the DIFFSYS 5.1 software, under the XZC interpolation mode, two types of D-shaped tool paths are simulated. When using the traditional D-shaped toolpath, the C-axis maintains a constant speed of 30 RPM in the transition zone and suddenly drops to 12 RPM upon entering the cutting zone. In contrast, with the optimized D-shaped toolpath, the C-axis speed undergoes a smoother change throughout the entire toolpath: it gradually decreases from 30 RPM in the transition zone to 19 RPM, and then further smoothly reduces from 19 RPM to 12 RPM in the cutting zone, before rising back to 19 RPM.

**Figure 8:**
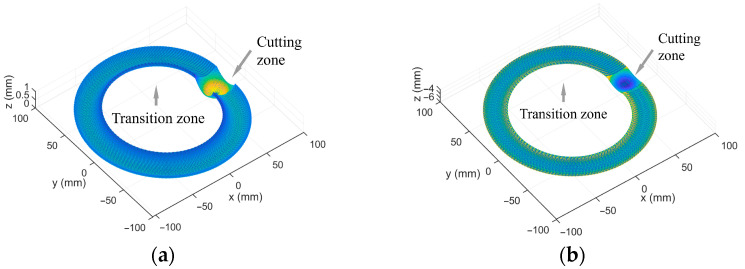
The three-dimensional D-shaped toolpath of the spherical surface and the integral mirror: (**a**) the D-shaped toolpath of the spherical surface; (**b**) the D-shaped toolpath of the integral mirror.

Based on the experience from actual processing, we adopt the following processing parameters. Due to the relatively large structural units of optical curved surface samples, the tool nose radius of the roughing and the finishing tools are 0.78 mm and 1.02 mm, respectively. As the material of the sample is aluminum, the rake angle of the single crystal diamond tool is 0° and the clearance angle is 12°. The eccentricity is 60 mm. The specific machining parameters of the two samples are shown in Table 1, and the 3D style of the linear toolpath is shown in Figure 8.

**Figure 9:**
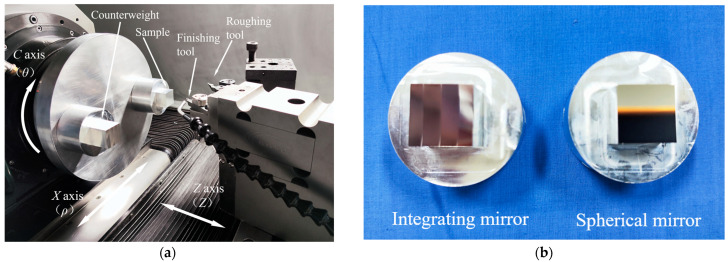
Actual turning lathe and samples: (**a**) turning lathe; (**b**) samples after turning.

Figure 9 shows the single point diamond turning machine (Moore Nanotech 250UPL, Swanzey, NJ, USA) used for processing samples in this experiment. The controller of this machine tool is NanoSmart V2 (Swanzey, NJ, USA). To reduce the dynamic balance error of the C-axis rotation, two samples are fixed on the chuck plate simultaneously. When processing one sample, the other can be used as a counterweight for the C-axis of the lathe.

The designed toolpath is used to complete the turning of spherical surface and then the turning of integral mirrors. The C-axis rotates clockwise, while the entire rotating mechanism moves in the X direction, and the Z-axis is the tool axis. The real-time monitoring system for programmable multi-axis controller shows that the servo following error remains within 7 nm throughout the processing. Figure 9b presents the spherical surface and the integral mirror processed in this experiment.

**Figure 10:**
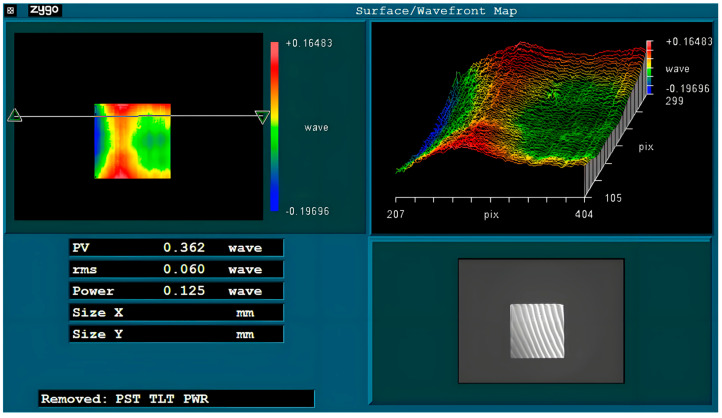
Zygo interferometer spherical detection image.

Figure 10 shows the Zygo interferometer detection map (Middlefield, CT, USA), wavefront error 0.361λ (PV, @632.8 nm). The overall accuracy has basically met the requirements of the non-imaging optical system, which verifies the feasibility of the D-shaped toolpath. In the wavefront map, faint vertical linear tool marks can also be observed, such as in the central area of the detection map. In this experiment, a processing step size of 10 μm was adopted. However, the feed-rate was too high, which affected the surface quality [31]. The lower cutting speed also causes the surface roughness to increase.

**Figure 11:**
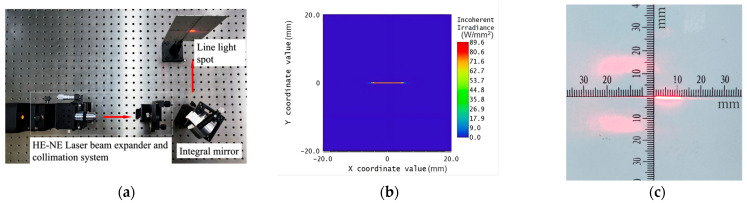
Integral mirror optical path: (**a**) integral mirror optical pat; (**b**) simulated light spot; (**c**) actual light spot.

Figure 11 shows the optical path of the integral mirror experiment, which is composed of the HE-NE laser collimation system (Tianjin Topus Instrument Co., Ltd., Tianjin, China), integral mirror and optical screen. The Gaussian circular beam with a diameter of 20 mm emitted from the laser collimation system is reflected by the integral mirror and forms a linear spot with a length of 10 mm at 300 mm. As shown in Figure 11b,c, the length of the linear spot is approximately 10.2 mm, with an error of no more than 2% compared to the theoretical length. In combination with the verification of spherical surface accuracy measurement, it is demonstrated that the optimized D-shaped toolpath proposed in this paper can be applied to the processing and manufacturing of cylindrical array optical surfaces.

## 4. Conclusions

The optimization design method of D-shaped toolpath proposed in this paper defines the toolpath in the transition zone with a fifth-order polynomial. In addition to effectively reducing the response frequency of the Z-axis linkage, the optimized toolpath can also precisely control the feed-rate and acceleration of the X-axis. The proposed method reduced the acceleration along the X-axis by 84%. Under the XZC interpolation mode, the optimized D-shaped toolpath enhances the stability of the C-axis spindle velocity, thereby improving the machining accuracy of off-axis optical surfaces. During the sample processing, the servo following error is within 7 nanometers, achieving surface accuracy of 0.361λ (PV, @632.8 nm). Within one processing cycle, one to four optical components can be processed simultaneously, significantly improving processing efficiency. This method not only reduces the servo-following error but also improves the processing efficiency. The approach provides a scalable solution for the production of off-axis optics such as linear gratings and cylindrical arrays.

## Figures and Tables

**Table 1:** Cutting parameters.

Parameters	Tool Nose Radius/mm	Eccentricity *d*_0_/mm	Cutting Zone/mm^2^	Angular Step Δ*θ*/°	Radial Step Size h/μm	Maximum Polar Angle in the Cutting Zone *θ*_1_/°	Depth of Cut/μm
Rough finish	0.78	60	22 × 22	0.5	100	8.8	20
Finishing	1.02	60	22 × 22	0.1	10	8.8	3

## Data Availability

The original contributions presented in this study are included in the article. Further inquiries can be directed to the corresponding authors.
